# Prenatal Allergen Exposure Perturbs Sexual Differentiation and Programs Lifelong Changes in Adult Social and Sexual Behavior

**DOI:** 10.1038/s41598-019-41258-2

**Published:** 2019-03-18

**Authors:** Kathryn M. Lenz, Lindsay A. Pickett, Christopher L. Wright, Anabel Galan, Margaret M. McCarthy

**Affiliations:** 1 Department of Psychology, The Ohio State University, Columbus, OH 43210 USA; 2 Department of Neuroscience, The Ohio State University, Columbus, OH 43210 USA; 3 Institute for Behavioral Medicine Research, The Ohio State University, Columbus, OH 43210 USA; 4 Department of Pharmacology, The University of Maryland School of Medicine, Baltimore, MD 21201 USA; 5 Program in Neuroscience, The University of Maryland School of Medicine, Baltimore, MD 21201 USA

## Abstract

Sexual differentiation is the early life process by which the brain is prepared for male or female typical behaviors, and is directed by sex chromosomes, hormones and early life experiences. We have recently found that innate immune cells residing in the brain, including microglia and mast cells, are more numerous in the male than female rat brain. Neuroimmune cells are also key participants in the sexual differentiation process, specifically organizing the synaptic development of the preoptic area and leading to male-typical sexual behavior in adulthood. Mast cells are known for their roles in allergic responses, thus in this study we sought to determine if exposure to an allergic response of the pregnant female in utero would alter the sexual differentiation of the preoptic area of offspring and resulting sociosexual behavior in later life. Pregnant rats were sensitized to ovalbumin (OVA), bred, and challenged intranasally with OVA on gestational day 15, which produced robust allergic inflammation, as measured by elevated immunoglobulin E. Offspring of these challenged mother rats were assessed relative to control rats in the early neonatal period for mast cell and microglia activation within their brains, downstream dendritic spine patterning on POA neurons, or grown to adulthood to assess behavior and dendritic spines. In utero exposure to allergic inflammation increased mast cell and microglia activation in the neonatal brain, and led to masculinization of dendritic spine density in the female POA. In adulthood, OVA-exposed females showed an increase in male-typical mounting behavior relative to control females. In contrast, OVA-exposed males showed evidence of dysmasculinization, including reduced microglia activation, reduced neonatal dendritic spine density, decreased male-typical copulatory behavior, and decreased olfactory preference for female-typical cues. Together these studies show that early life allergic events may contribute to natural variations in both male and female sexual behavior, potentially via underlying effects on brain-resident mast cells.

## Introduction

Sexual differentiation of the rodent brain occurs during a narrow developmental window that begins prenatally and extends into the early postnatal period. During this “critical period” males are exposed to high levels of androgens that are derived from the testes, and these androgens are converted to estrogens in the brain and subsequently direct brain development in a male-typical pattern^1^. In the absence of steroid hormones, the brain develops in a female-typical pattern. Sex differences in brain development prepare the brain to direct sex-specific behavioral repertoires necessary for successful reproduction. The preoptic area (POA) is a brain region responsible for both the motivational and consummatory aspects of male sexual behavior^2^. Our lab and others have focused on how perinatal hormone exposure leads to male-typical development of the POA in rats. Several of the downstream effectors of hormonally-driven sexual differentiation have been identified, and one of the key players of this process is the brain’s immune system^3^.

Microglia, the primary resident immune cells of the brain, are both targets and effectors of the sexual differentiation process. Males have twice the number of ameboid-shaped microglia in the POA as a result of estradiol exposure in early life^4^. This higher microglia load leads to higher levels of the pro-inflammatory lipid, prostaglandin E2 (PGE2) in the male compared to the female POA^4^. PGE2 in turn is responsible for establishing a higher density of dendritic spine synapses in the developing male POA^5^ which persist into adulthood and positively correlate with the display of male copulatory behavior^6^. We have recently discovered that another innate immune cell type within the brain, the mast cell, is also a target and effector of sexual differentiation^7^.

Mast cells are tissue resident granulocytic innate immune cells that are activated by exposure to allergens^8^. They are distributed throughout the body, mostly at interfaces, but also reside in the brain. They are found inside the blood-brain-barrier but typically cluster at the meninges^9^. We have found that mast cells are more numerous and more activated within the neuropil of male rat POA during perinatal brain development, and that estradiol acts directly on these mast cells to stimulate the release of histamine^7^. This histamine is in turn sufficient to activate neighboring microglia and set off the cascade of microglia activation and production of PGE2 that drives male-typical dendritic spine patterning in the POA. In this way, the immune system is indispensable for brain sexual differentiation. In females, pharmacologically activating mast cells leads to masculinization of dendritic spine patterning in the POA as well as the masculinization of copulatory behavior^7^, suggesting that mast cell activation via non-pharmacological means could shift female sexual development toward a masculinized phenotype.

During this same critical period for brain sexual differentiation, perinatal exposure to many perturbations can independently alter the trajectory of brain development by activating immune cells in the brain. These include stress, infection, inflammation, over or undernutrition, or hypoxia. Maternal conditions or illnesses, such as bacterial and viral infections, preeclampsia, autoimmune disorders, asthma, or allergies, can similarly shape offspring brain and behavioral development^10–12^. But to date, the effects of such early life inflammatory perturbations on sexual differentiation and the establishment of sex differences in brain and behavior have not been explored. Given that our previous studies have indicated that mast cells and microglia are integral to sexual differentiation, we sought to determine whether this essential developmental process is sensitive to prenatal allergic inflammation. We report herein that allergic challenge of pregnant dams impacted the sexual differentiation of both the male and female POA, inducing masculinized brain and behavioral development in female offspring while also dysmasculinizing brain and behavioral development in male offspring.

## Materials and Methods

### Animals

All experimental procedures were approved by the Institutional Animal Care and Use Committees at either the University of Maryland School of Medicine or The Ohio State University, and followed all guidelines put forth in the Guide for Care and Use of Laboratory Animals. Sprague Dawley rats purchased from Harlan Laboratories or bred in-house from animals originally purchased from Harlan. Animals were housed on a reversed 12 h light/dark cycle in standard group cages, except when breeding, with food and water ad libitum. Adult females were paired with males and separated when vaginal lavage was sperm-positive. Once sperm-positive, pregnant females were isolated and allowed to deliver naturally. Rat pups used in experiments were birthed in-house, and pups from multiple litters were randomly assigned to experimental treatment and then randomly distributed back to dams to control for differences in maternal care. Cages were checked daily to determine the day of birth (designated postnatal day (PN) 0).

### Intraperitoneal injections

Lipopolysaccharide from *E. coli* (LPS; strain K-235, cat#L2143, Sigma; dose: 200 μ/kg ip in 0.05 ml pyrogen-free saline) was given as an immune challenge via intraperitoneal injection on PN0, and control animals received an equivalent injection of saline vehicle. LPS-challenged animals were euthanized on PN4 and tissue collected for western blot (detailed below).

### Gestational allergic challenge

Prior to pregnancy, adult females assigned randomly to the experimental group were sensitized with a subcutaneous injection of 1 mg ovalbumin (OVA grade V, Sigma) prepared at 4 mg/ml in pyrogen-free 0.9% saline and precipitated at a 1:1 ratio with Al(OH)_3_ (Thermo Scientific) according to manufacturer’s instructions. After two weeks, a second 1 mg ovalbumin-Alum adjuvant injection was given. Control females were injected with saline at the same two timepoints to control for experimental handling and stress effects. One week later, all females were paired with males for breeding and the day of detection of sperm assigned gestational day 0 (GD0). At GD15, pregnant rats were challenged intranasally with 1% ovalbumin in saline (experimental group) or saline vehicle (control group) (volume: 50 µl per nare), which was placed on each nare under light isoflurane anesthesia and inhaled upon regaining consciousness. At 30 min following challenge, maternal blood was collected to assay for total serum Immunoglobulin E (IgE), using an IgE Rat ELISA kit (Abcam cat#157736) and serum samples run in triplicate. Females were paired in groups of two until GD15 and then housed individually. After birth, animals were sacrificed via perfusion to analyze brain-resident mast cells, microglia or neuronal morphology at specified time points, or were weaned at PN22 into sex-specific groups of three containing both OVA challenged and vehicle exposed offspring for behavioral testing.

### Histology and immunohistochemistry

For all *in vivo* histology experiments, animals were killed via lethal overdose with FatalPlus (Vortech Pharma) followed by transcardial perfusion with saline followed by 4% paraformaldehyde, brains removed, and postfixed for 12 hours. Brains were sectioned coronally at 45 μm thickness on a cryostat (Leica) and mounted onto SuperFrost charged slides (Fisherbrand) for subsequent staining procedures.

#### Mast cell staining

Mast cells were visualized using staining with acidic Toluidine Blue (Sigma; 0.5% in 60% ethanol; pH = 2.0; 10 minute stain incubation) as detailed in^7^, and then tissue was cleared with ascending ethanol, defatted with xylenes, and coverslipped using Permount.

#### Immunohistochemistry (IHC)

Brain sections were rinsed twice with PBS, permeabilized with 0.3% H_2_0_2_ in 50% methanol, blocked with 5–10% bovine serum albumin or normal goat serum in PBS + 0.4% Triton X, and incubated with primary antisera against the pan-microglia marker, Iba1 (Wako cat#019-19741 1:1000) for 24 hours at 4 °C. Sections were extensively washed, and processed with biotinylated secondary antibodies (Vector), avidin-biotin complex (Vector), and reacted with Nickel-diaminobenzidine in 0.125 M sodium acetate to visualize chromogen for IHC. Stained sections were coverslipped with DPX mounting medium.

### Golgi-Cox staining

Whole brains from P5 pups or P80–90 adults were placed in 15 ml of Golgi-Cox solutions A and B (FD Neurotech) for 10 days, then solution C (sucrose; FD Neurotech) for 1–1.5 weeks, cut into coronal sections 100 µm thick using a Leica vibrotome, and staining embedded using solutions D + E following the FD Neurotech protocol. Tissue was cleared with ascending ethanol, defatted with xylenes, and coverslipped using Permount.

### Microscopy and Stereology

Stereology and single cell reconstruction: A Zeiss Axioimager.M2 microscope coupled to a CX9000 Digital Camera and Stereo Investigator software (MBF Bioscience) were used to estimate the total population of mast cells and microglia in the POA, using an average of 4–6 sections per animal encompassing the entire rostrocaudal extent of the POA. At the time of counting, mast cells were categorized as either granulated or degranulated, and microglia were categorized as ameboid or not ameboid. Microglia were considered ameboid if they had an enlarged cell body and either no processes or few, short processes, based on criteria validated in our previously published study^4^.

For analysis of Golgi-Cox impregnated POA neurons, neurons were chosen for analysis if the cell body was in the middle 50% of the z plane of the tissue section, multiple processes were visible and the cell was easily distinguishable from nearby cells. Four-to-five cells per animal across 3–4 brain sections were reconstructed in three dimensions under a 100x oil objective using Neurolucida software (MBF Bioscience). Morphological parameters for each cell were computed using Neurolucida Explorer, including cell body size, total dendritic length per cell, number of dendritic segments and branch points, and total number of dendritic spines per neuron. Data presented is the average of each of these parameters across multiple neurons for each animal.

For adult analyses, dendritic spines were analyzed, but single cells were not reconstructed in three dimensions. Dendritic segments chosen for analysis were unobstructed by other Golgi-stained material, and were at least 25 μm in length without a bifurcation. Only one segment was analyzed per given cell. Four-to-five dendritic segments per animal across multiple brain sections were reconstructed, and dendritic spine density was analyzed using Neurolucida Explorer software. Data are presented and were analyzed using the average dendritic spine density for each animal.

### Western blot

Tissue was homogenized in RIPA buffer containing 1% Igepal CA630, 0.25% deoxycholic acid, 1 mM EDTA, 154 mM NaCl, and 65 mM Trizma Base, with added protease and phosphatase inhibitors (1:1000). All chemicals were obtained from Sigma unless otherwise specified. Protein supernatant was extracted after 20 minutes of centrifugation at 3000 rpm at 4 °C, and total protein concentration determined via Bradford assay (BioRad). Fifteen μg of protein was electrophoresed on an 8–16% precast SDS polyacrylamide gel (Life Technologies) and transferred onto a single polyvinyl difluoride membrane (Bio-Rad). Membranes were blocked in 50% Odyssey blocking buffer (LI-COR) in TBS or 10% nonfat milk in 0.1% Tween in Tris-buffered saline (TTBS) and subsequently incubated with spinophilin primary anti-serum (Millipore cat#06–842, 1:1000) in 5% milk in TTBS overnight at 4 °C. Membranes were rinsed and incubated with HRP-conjugated secondary antibody (1:200) for two hours. A Phototype chemilluminescence system (New England Biolabs) was used to detect the immunoblots by exposing the membrane to Hyperfield ECL (GE Healthcare). Integrative grayscale pixel area densitometry captured with a CCD camera was quantified with NIH Image software. Ponceau S staining appearing at 45 kDa was used as a loading control, and immunoblot densitometry values for each lane expressed as a percentage of Ponceau staining for the same lane.

### Behavior

#### Sexual behavior testing

Between PN50–54, animals were gonadectomized under isoflurane anesthesia and implanted subcutaneously with a 30-mm silastic capsule (1.57 mm inner diameter, 3.18 mm outer diameter) filled with crystalline testosterone (Sigma) placed between the scapula. This capsule length releases testosterone in a manner that mimics physiological levels of testosterone circulating in adult males and allows appropriate activational hormones for developmentally-masculinized females to perform male-typical copulatory behavior^5^. Two weeks following surgery, animals were video recorded for at least 20 min during the dark phase of the light cycle, in a Plexiglass behavioral arena in the presence of a hormonally-primed receptive stimulus female under red light illumination. Behavioral data was collected and analyzed by an observer blind to the experimental treatment of each animal. Measures included number of mounts, latency to mount, frequency of ejaculation, time of each ejaculation and latency to resume mounting after ejaculation (ejaculation measures in males only). In order to record the full post-ejaculatory interval for situations during which a male started a post-ejaculatory interval with only a portion of the 20 min testing period remaining, the male was observed beyond the 20 min period until he started mounting again. These mounts outside of the 20 min period were not tallied in total mount measures. Mount rate was calculated from the total mounts divided by the 20 minutes less the total time during that 20 min the animal was in a refractory post-ejaculatory state.

#### Olfactory preference test

Olfactory preference test was performed on a separate cohort of adult animals (~PN60) from those used for sexual behavior testing. The test was performed using a modified protocol based on^13^. Animals were placed in the same Plexiglas arena used for sexual behavior testing, with two ceramic dishes in opposite corners of the arena (counterbalanced across groups and animals). Dishes contained soiled bedding from adult male or female non-littermate conspecifics’ cages, freshly collected 5 days after bedding change. Animals were placed in the center of the arena and allowed to openly explore the arena for 5 min under red light illumination during the dark phase of the light cycle, while being videotaped. The number of seconds spent actively investigating (e.g., sniffing, digging in, or climbing on/in) each dish of soiled bedding was tallied and a female bedding preference score tabulated ([Time spent investigating female bedding- time spent investigating male bedding]/total investigation time).

### Data analysis

All histological, immunohistochemical, and western blot data were analyzed using one- or two-tailed t-tests or one or two-way ANOVAs if normally distributed, followed by Tukey’s HSD post-hoc analysis. When not normally distributed, behavior data were analyzed by Mann-Whitney U tests correcting α-significance for family-wise error. Overall statistical significance was set at α = 0.05. Exact p-values are presented in figure captions for each dataset and analysis. Effect sizes (*r* for t-tests and η^2^_p_ for ANOVA) are reported along with exact statistic values (t or F) and degrees of freedom for each effect. All data were analyzed using GraphPad Prism or SPSS software, and all graphs made using GraphPad Prism. Statistical results are presented in the text and group sizes for each experiment are presented in each figure caption.

## Results

**Figure 1:**
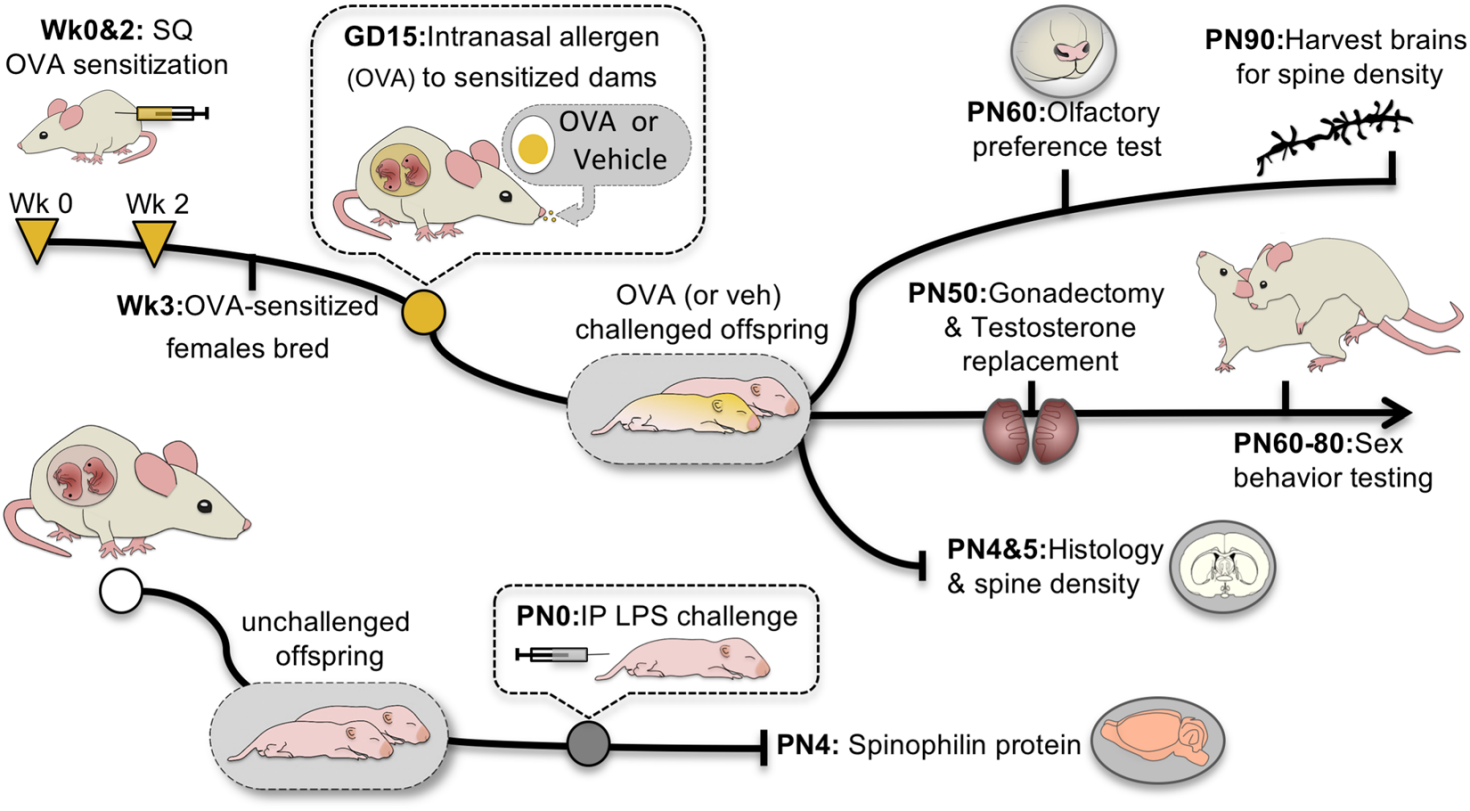
Prenatal allergic and postnatal immune challenge models. Ovalbumin (OVA) sensitized or control females were bred and challenged intranasally with OVA or vehicle on gestational day (GD) 15, and pups assessed neonatally for brain-resident immune cell numbers or grown to adulthood for sociosexual behavior testing and neuroanatomical assessment. Neonatal offspring from naive dams were challenged on the day of birth with an intraperitoneal injection of lipopolysaccharide (LPS), and the POA assessed on PN4 for spinophilin protein content.

**Figure 2:**
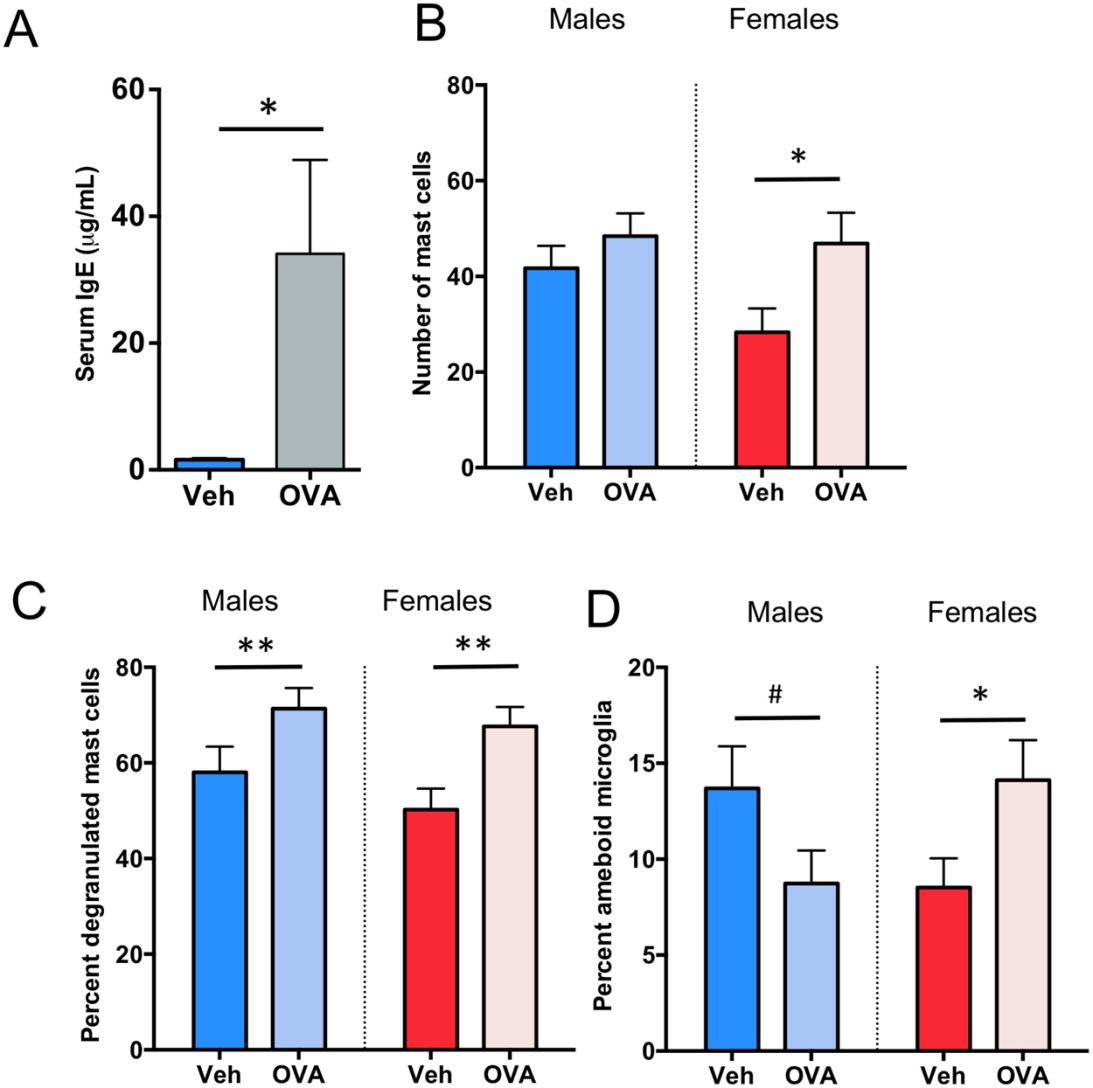
Effects of prenatal allergic challenge on mast cells and microglia in the POA. (**A**) Intranasal administration of OVA to pregnant dams at GD15 increased serum IgE levels in dams, confirming an allergic response. (**B**) Exposure to prenatal allergic challenge increased mast cell number in the POA of newborn females relative to controls. (**C**) Allergic challenge also increased the percent of mast cells that were degranulating on PN4 in both sexes, (**D**) but impacted microglia in males and females differently. Allergic challenge increased the percentage of ameboid microglia in the female POA relative to controls and there was a trend toward a significant decrease in ameboid microglia in males. Data presented as mean + SEM. *Indicates p < 0.05, **indicates p < 0.01, ****indicates p < 0.0001, ^#^indicates trend 0.05 ≤ p < 0.1. Group sizes: Panel (A). All groups n = 3. Panel (B,C): ♂V n = 10, ♂OVA n = 15, ♀V n = 15, ♀OVA n = 14. E: ♂V n = 4, ♂OVA n = 7, ♀V n = 8, ♀OVA n = 6.

To determine whether a physiologically relevant allergen-based immune challenge could influence sexual differentiation of the POA and resulting behavior, we developed an allergic challenge model that induces IgE production in maternal dams. Adult female rats were sensitized to the allergen ovalbumin (OVA), bred, and challenged intranasally with OVA in saline or saline vehicle on gestational day (GD) 15 of pregnancy (Fig. 1), which induced a significant acute IgE response in maternal dams (t_(4)_ = 2.20, *p* = 0.046; *d* = 0.74; Fig. 2A). When offspring were assessed on postnatal day (PN)4, we found that gestational allergic challenge significantly increased the number of mast cells in the female POA to male-typical levels (F_(1,50)_ = 5.36; *p* = 0.025; η^2^_p_ = 0.097; Fig. 2B). Prenatal allergic challenge also increased the proportion of activated, or degranulated, mast cells in both males and females (F_(1,50)_ = 10.73, *p* = 0.002; η^2^_p_ = 0.204; Fig. 2C). This gestational allergic challenge led to postnatal consequences for microglia too, that differed by sex (F_int.(1,21)_ = 7.76, *p* = 0.01; η^2^_p_ = 0.270; Fig. 2D). In females, allergen exposure led to an increased percentage of microglia with ameboid shaped morphology in females to male-typical levels (HSD p = 0.03; Fig. 2D). There was a trend towards the opposite effect in males with a reduction in the percent of ameboid shaped microglia (HSD p = 0.09; Fig. 2D), suggesting that at least on the short-term the response to in utero allergic challenge is opposite in males and females.

**Figure 3:**
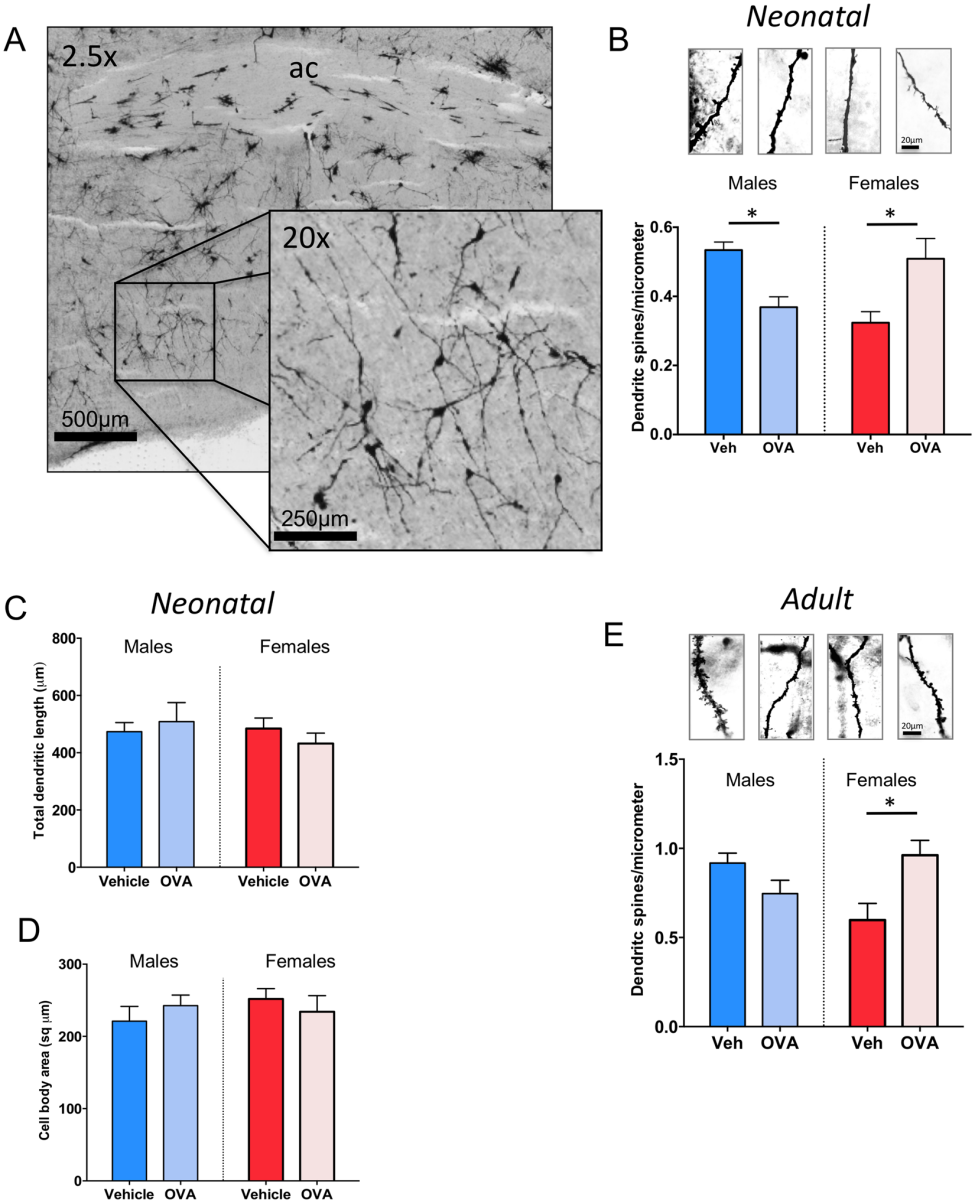
Prenatal allergic challenge and mast cell induced masculinization of POA dendritic spines and spinophilin *in vivo*. (**A–D**) Prenatal allergic challenge increased the density of dendritic spines in Golgi-Cox impregnated POA neurons of females on PN5, and decreased the density on male POA neurons, but had no effect on total dendritic length (**C**) or cell body area (**D**). (**E**) Prenatal allergic challenge led to lifelong masculinization of dendritic spine density of females relative to vehicle females, as measured in adult POA neurons visualized with Golgi-Cox impregnation, but had no enduring effect on males. Group sizes: Panel (B–D). All groups n = 4. E: ♂V n = 4; ♀V n = 3; ♂V n = 4; ♀OVA n = 5.

**Figure 4:**
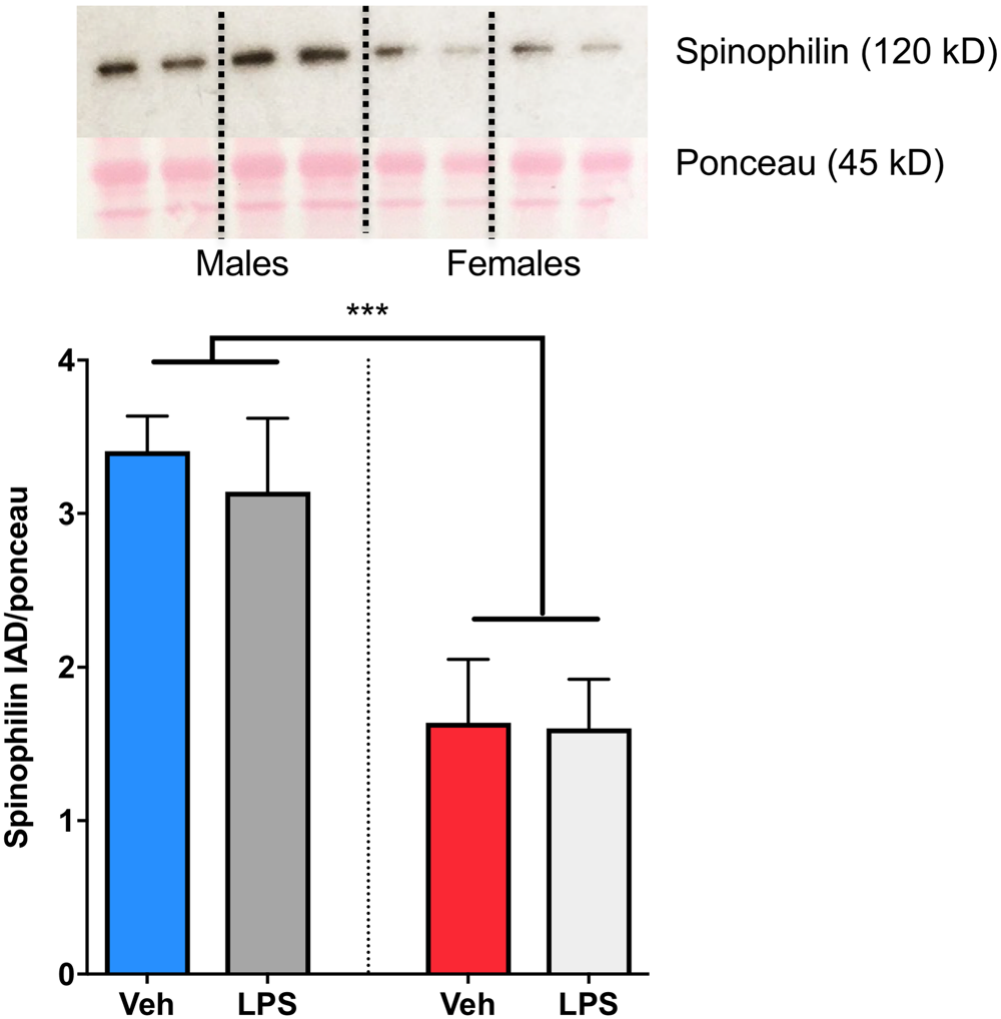
No Effect of LPS immune challenge on spinophilin content in the POA. A well established sex difference in POA spinophilin content as measured by western blot was observed. However, immune challenge with lipopolysaccharide (LPS) on the day of birth had no effect on spinophilin content in the POA of males or females on PN4. ***Indicates p < 0.001. Group sizes: ♂V n = 5; ♂LPS n = 5, ♀V n = 7, ♀LPS n = 7. Western blot representative images are cropped from a single location on the full length gels. Full length images can be viewed in Supplementary Fig. 1.

Neurons in the POA are sexually dimorphic, with males having 2–3 times the density of dendritic spine synapses as females^5^. The density of dendritic spine synapses correlates positively with the display of male-typical copulatory behavior in adulthood. We previously showed that pharmacological activation of mast cells in neonatal females led to increased markers for dendritic spines as well as increased masculine copulatory behavior in adulthood^7^. Here, we found that neonatal females gestated in an allergic-challenged dam had male-like dendritic spine density on Golgi-Cox impregnated POA neurons, while neonatal males from those same dams exhibited signs of dysmasculinization as density of dendritic spines was reduced (F_int(1,12)_ = 20.92, *p* = 0.0006; η^2^_p_ = 0.64; HSD post-hoc p’s < 0.05; Fig. 3A,B). There was no sex difference or effect of allergic challenge on overall dendritic length (F_int(1,12)_ = 0.94 p = 0.351; F_treat(3,16)_ = 0.035, p = 0.855; F_sex_ = 0.538 p = 0.478; Fig. 3C) or the size of the soma (F_int(3,22)_ = 1.167 p = 0.301; F_treat(3,16)_ = 0.10, p = 0.921; F_sex_ = 0.379 p = 0.550; Fig. 3D). The masculinization of dendritic spine density observed in neonatal females endured into adulthood, but the decrease observed in neonatal males was no longer evident in adulthood, with males gestated in allergic-challenged dams being indistinguishable from control males (F_int(1,12)_ = 11.24, p = 0.006, η^2^_p_ = 0.45; Tukey’s HSD p = 0.016, Fig. 3E). While Golgi-Cox impregnation allows for the counting of spine heads and a measure of spine density, we have found quantification by western blot of the protein spinophilin to be a reliable proxy marker for the amount of dendritic spines on POA neurons, consistently revealing more spinophilin in male POA than female^3,4,7,14,15^. We here again used spinophilin as a proxy marker and detected a robust sex difference (F_(1,20)_ = 18.62, p = 0.0003; η^2^_p_ = 0.48). However, in contrast to the masculinizing effects of prenatal allergen exposure on dendritic spines, immune challenge with the bacterial endotoxin, lipopolysaccharide (LPS) did not lead to masculinization of spinophilin when measured by western blot on POA tissue from PN4 pups (F_(1,20)_ = 0.16, p = 0.69; Fig. 4), indicating some specificity of allergic inflammation in particular on the masculinization process in the POA.

**Figure 5:**
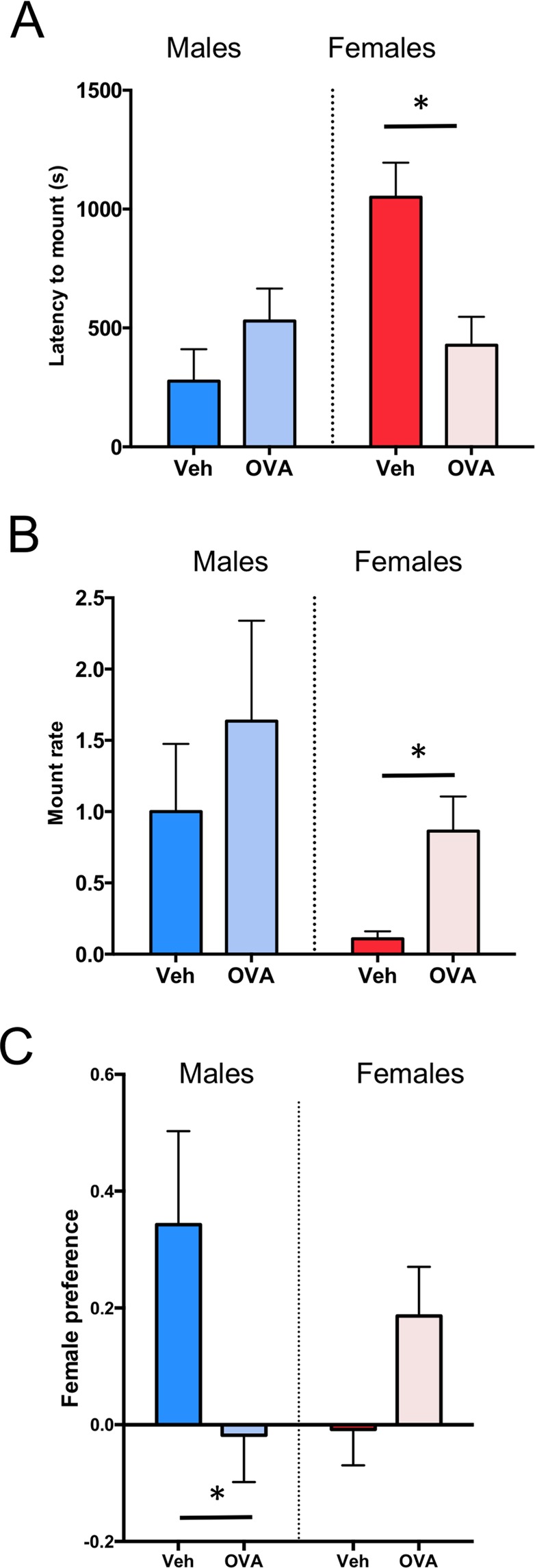
Effects of prenatal allergic challenge on male-typical reproductive behaviors in adulthood. Adult females exposed to a maternal allergic challenge *in utero* began mounting more quickly (**A**) and mounted more frequently (**B**) than control females such that they were indistinguishable from control males. (**C**) In olfactory preference testing, males showed a significant bias in odor preference compared to female counterparts; however, odor preference was abolished in males experiencing allergic challenge *in utero* relative to unchallenged males. Allergic challenge *in utero* had no significant effect on odor preference of female littermates (p = 0.2). Data presented as mean + SEM. *Indicates p < 0.05. Group sizes: A-B: ♂V n = 6, ♂OVA n = 9, ♀V n = 5, Ova ♀ n = 9. C: ♂V n = 7, ♂Ova n = 7, ♀V n = 6, Ova ♀ n = 7.

We next sought to determine if mast cell activation in developing females via allergic challenge is sufficient to induce behavioral masculinization. Using the same prenatal allergic challenge model, we found that females exposed to a prenatal allergic challenge and then treated with testosterone in adulthood in order to assess their capacity for male sexual behavior, started mounting a stimulus receptive female more quickly (M-W U = 45, p = 0.013; Fig. 5A) and mounted more often (M-W U = 5, p = 0.013; Fig. 5B) than non-exposed females, reflecting a high degree of motivation and behavioral execution in the OVA allergen exposed females. Thus, the same allergic challenge that suffices to recruit mast cell numbers and induce degranulation to male levels (Fig. 2), also masculinizes sexual behavior. Generally, males prefer to investigate bedding soiled by rats of the opposite sex whereas females generally show no bias in preference, unless they are in estrus. In olfactory preference testing, we found a sex by treatment interaction (F_(1,23)_ = 6.74, p = 0.02; η^2^_p_ = 0.18; Fig. 5C). The preference for female odors exhibited by control males was abolished in those experiencing allergic challenge *in utero*, Tukey’s HSD p = 0.02), whereas there was no impact on odor preference by females with or without OVA treatment.

Overall, these data are consistent with the view that mast cells are crucial regulators of early life programming of adult sex-specific behavioral repertoires, and suggest that both prenatal and postnatal effects of mast cells contribute to sex-specific behavioral programming. However, additional components of the allergic response may contribute to dysmasculinization in males and/or masculinization of females.

## Discussion

Brain sexual differentiation is primarily directed by the action of steroid hormones, such as androgens and estrogens during a perinatal critical period. We have recently demonstrated that a major target of steroid hormone action in the developing preoptic area are cells derived from the immune system, called mast cells. Mast cells are more numerous in the male POA and are activated by estradiol. Furthermore, mast cells jumpstart the masculinization of synaptic connectivity in the POA by releasing histamine, which activates neighboring microglia to release the spinogenic factor, PGE2^7^. Pharmacological activation of mast cells in females leads to neuroanatomical and behavioral masculinization, and conversely, pharmacological inhibition of mast cells leads to dysmasculinization of the POA and subsequent sexual behavior in males^7^.

In the current studies, we sought to determine whether a physiologically-relevant mast cell activation event during gestation, namely maternal allergen exposure, could likewise perturb sexual differentiation of the POA and resulting sociosexual behaviors later in life. The current results build upon our previous pharmacological studies and together suggest that mast cells basally contribute to the masculinization process in males. We found that allergic inflammation in pregnant dams resulted in increased mast cell number in the POAs of female offspring, and mast cell activation in the brains of both male and female offspring. The prenatal allergic inflammation in our model occurred during mid-gestation, at GD15, and offspring brains were assessed for mast cell and microglia in the early postnatal period following birth. We found that both mast cell number and degranulation were increased and microglia morphology showed a shift toward a reactive phenotype in the early postnatal brains of female offspring, indicating that allergen-induced inflammation endured for over a week, and may in fact endure for even longer, though we did not assess the full duration of the inflammation induced in the current study. Regardless, prenatal allergic inflammation had long-term consequences for the sexual differentiation process in both males and females, impacting their behavior in adulthood. Prenatal allergen exposure in females shifted them towards a male-typical organization of synaptic patterning in the neonatal POA that endured into adulthood. In contrast, males that were prenatally exposed to allergic inflammation showed evidence of dysmasculinization, including reductions in dendritic spine synapses in the POA as well as decreased mounting behavior and female olfactory preference in adulthood. This dysmasculinization may reflect an inverted U shaped effect of immune signaling in the male brain, such that too little inflammatory signaling would lead to feminization of brain development, but excessive activation of the immune system in males by exogenous inflammatory insults pushes male neuroimmune activation beyond the optimal level for masculinization and thus perturbs the masculinization process.

Interestingly, postnatal exposure to inflammation in the form of immune challenge with LPS, had no effect on basal sex differences in the dendritic spine marker, spinophilin. This may be because a postnatal immune challenge is too late to perturb sexual differentiation of the POA which begins prenatally, but many masculinizing agents given to postnatal females are effective, including a single intracerebral injection of PGE2^6^. A more likely explanation is an incomplete response to LPS by the immature POA, as seen for other brain regions^16^. In this study it was the allergic reaction of the mature dam that impacted the developing fetuses and it is unknown if LPS given during the same period of gestation would have a similar impact. Future studies will be necessary to determine the extent to which allergic inflammation is distinct from classic endotoxin immune challenges, such as with LPS.

Mast cells reside in the brains of rodents and humans and are more abundant in the immature versus adult brain^7,17^. This suggests that the perinatal period may be a particular window of vulnerability for pathological mast cell activation following exposure to allergic inflammation. Ours is the first study to assess the response of mast cells to maternal immune activation of any variety. While the current studies do not demonstrate a causal relationship between increased mast cell number and activation in the rodent brain and resulting changes in sexual behavior, these findings coupled with our previous mechanistic work linking mast cells to sexual differentiation via targeted pharmacological manipulations strongly support the conclusion that allergen-induced effects on sex-typical neural and behavioral development are driven by mast cells.

Recent studies using rodents indicate that chronic allergic asthma during pregnancy alters offspring behavioral development, including social interaction and anxiety behaviors^18,19^. A chronic gestational allergic asthma rodent model found microglia gene expression was significantly altered in the offspring brain, and gene sets associated with autism were particularly affected^20^. These studies were only performed in females, and thus effects of chronic allergic inflammation during pregnancy on males is unknown. We found that a single timed allergic event is sufficient to impact sociosexual behaviors in both male and female offspring months following the allergen exposure. Though males and females were both impacted by prenatal allergic inflammation, the directionality of the effects on both synaptic patterning and sociosexual behavior were opposite. Males have more microglia and mast cells within the developing rat brain^4,7^, but the fact that we see differences in the directionality of early life allergic inflammation on males and females raises the possibility that sex differences not only in the number of immune cells in the brain, such as mast cells and microglia, but also in the phenotype and behavior of these cells, may contribute to sex-specific effects of early life inflammation.

While it is clear that prenatal allergic inflammation leads to the activation of mast cells within the offspring brain, the mechanisms through which the inflammatory stimulation is conferred to the fetal compartment remains unknown. Maternal immune activation or chronic allergic inflammation increases maternal circulating cytokines and placental cytokine production^18,21,22^. We demonstrated that maternal IgE is significantly elevated following allergic inflammation, and we have previously shown that mast cells in the offspring brain express the receptor for IgE, FcεR1^7^. In humans, IgG was long thought to be the only maternal immunoglobulin to cross the placenta into the fetus, but IgE has been detected in the fetal portion of the placenta^23,24^ and in cord blood^25^, indicating that IgE can enter the fetal compartment and thereby influence fetal development. We do not know the degree of protection from maternal inflammatory effectors the placenta offers the fetal compartment at this period in development, thus additional studies are required to attribute fetal mast cell activation to elevated maternal IgE and/or cytokines. In adult rats, exposure to allergens following allergic sensitization leads to an elevation of IgE in the brain^26^, though the mechanisms through which this occurs or the relevance of immunoglobulin increases in the brain to behavioral outcomes is unknown. Interestingly, in adult mice, allergic inflammation leads to acute increases in mast cell numbers in males, but not females^27^. Mast cells can be recruited into the brain by injury or autoimmune processes, and this sometimes occurs in a sex-specific manner^28,29^.

The source of sex differences in mast cells in the brain is unknown. The mast cells in the neonatal rat POA express estrogen receptor alpha, and estradiol may induce recruitment of mast cells into the brain given that brain-resident mast cell numbers increase in females within days following estradiol treatment. In a preliminary analysis, neither males nor females show evidence of proliferating mast cells in the neonatal POA^7^. Previous work in rodents and birds reveal mast cell numbers in the brain are dynamic across reproductive conditions, increasing within the habenula during mating in doves^30^, and in somatosensory areas of the thalamus of female rats during estrus^31^. Thus mast cells may be modulated by sex specific signals, either hormonal or behavioral, throughout life. Interestingly, a recent transcriptomics study of mast cells from the gut uncovered dramatic sex differences in gene expression^32^, but the hormonal dependence of these differences remains unknown. It is likely that sex differences in mast cells serve other functions beyond brain sexual differentiation and that sex differences in physiology throughout the body that are modulated by mast cells.

Female brain development is considered the “default” because it occurs without the need for gonadal steroid signaling. As a genetically programmed process, female typical development should therefore occur along highly similar lines across individuals. And yet, behavioral phenotypes across the female population are more variable than in the male population^33^. For example, tomboys are a common variation in female behavior that has not been explained by developmental androgen exposure^34^. Similarly, women with congenital adrenal hyperplasia (CAH), who experience high fetal androgen levels show wide variability in the degree to which they exhibit behavioral masculinization and this does not correlate with the highly-androgen dependent degree of genital masculinization^35^. This dissociation between genital and behavioral masculinization in CAH women underscores that behavioral outcomes are controlled by many factors besides hormonal exposure. In this study, we found that a relatively common early life experience, exposure to allergic inflammation and downstream activation of mast cells, shifts the trajectory of female behavioral development toward a masculinized phenotype. It is important too to note that the shifts in behavior seen in our female rats are all within the ‘normal’ range, and thus may be reflective of variability in female behavior that is seen in humans. Our study suggests that a heretofore unknown source of the observed variability in female behavior could originate with mast cells.

These findings illustrate that immune cells are involved in the process of brain sexual differentiation, and that prenatal allergic inflammation perturbs this crucial process in both sexes. Since many mental health and neurological disorders show a sex bias in prevalence^3^, it is possible that inflammatory events early in life may influence males and females differently due to underlying sex differences in the neuroimmune system.

## Supplementary information

Supplementary Figure 1
