# Supplementary material for: Prenatal Allergen Exposure Perturbs Sexual Differentiation and Programs Lifelong Changes in Adult Social and Sexual Behavior
Source: Sci Rep. 2019 Mar 18;9:4837. doi: 10.1038/s41598-019-41258-2 (PMC6423032; doi:10.1038/s41598-019-41258-2)
Supplement: Supplementary file 1 — Supplementary Figure 1 [file 41598_2019_41258_MOESM1_ESM.pdf]

Title: Prenatal Allergen Exposure Perturbs Sexual Differentiation and Programs Lifelong Changes in Adult Social and Sexual Behavior

Authors: Kathryn M. Lenz, Lindsay A. Pickett, Christopher L. Wright, Anabel Galan, Margaret M. McCarthy

Supplemental Figure 1

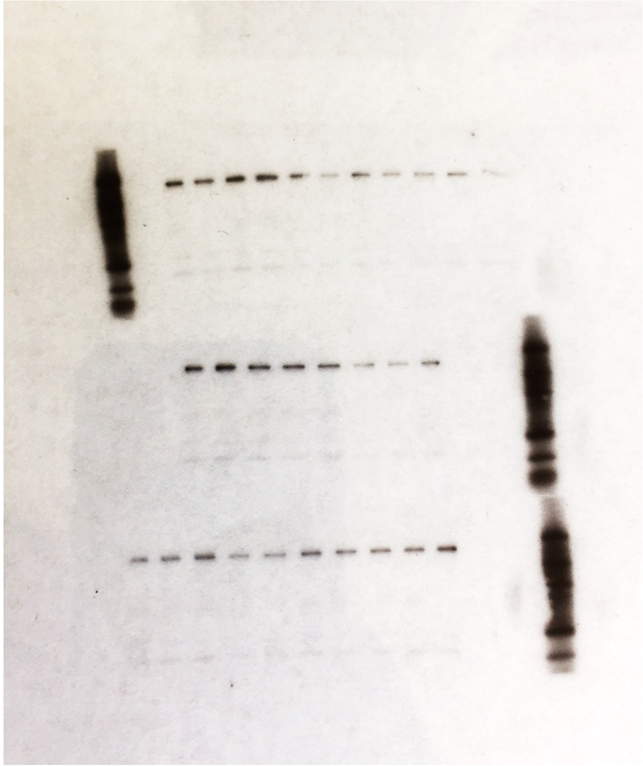

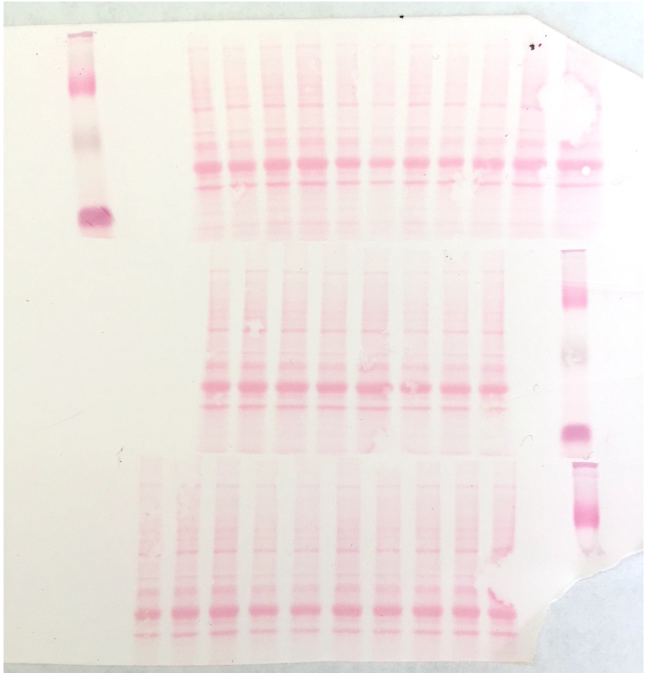

Supplemental figure 1: Full length western blot for spinophilin (upper) and Ponceau staining of the same membrane (lower) for data in Figure 4. The first 8 lanes were used for representative images in Figure 4.
